# Phylogenetic relationship and characterization of the complete mitochondrial genome of *Camponotus japonicus* (Hymenoptera: Formicoidea: Formicidae)

**DOI:** 10.1080/23802359.2022.2067495

**Published:** 2022-04-22

**Authors:** Shuo Shen, Wei Li

**Affiliations:** a Academy of Agriculture and Forestry Sciences, Qinghai University, Xining, China; b State Key Laboratory of Plateau Ecology and Agriculture, Qinghai University, Xining, China; c Scientific Observing and Experimental Station of Crop Pest in Xining, Ministry of Agriculture, Xining, China; d Key Laboratory of Agricultural Integrated Pest Management of Qinghai Province, Xining, China

**Keywords:** Formicidae, *Camponotus japonicus*, mitochondrial genome, phylogenetic analysis

## Abstract

*Camponotus japonicus* Mayr, 1866 is a widespread and open-field formicine ant species in east Asia. In this study, we sequenced and analyzed the complete mitochondrial genome (mitogenome) of *C. japonicus*. This mitogenome was 16,422 bp long and encoded 13 protein-coding genes (PCGs), 22 transfer RNA genes (tRNAs) and two ribosomal RNA unit genes (rRNAs). Gene order was conserved and identical to most other previously sequenced Formicidae. All PCGs of *C. japonicus* have the conventional start codon for invertebrate mitochondrial PCGs (five ATT, four ATA and four ATG). All PCGs terminated with the stop codon TAA. The whole mitogenome exhibited heavy AT nucleotide bias (77.8%). Phylogenetic analysis positioned *C. japonicus* in a well-supported clade with *C. atrox* and *C. concavus*, and *C. japonicus* was more closely related to *C. atrox* than to *C. concavus*. Within Formicinae, the topology (*Colobopsis* + (*Polyrhachis* + *Camponotus*) + (*Formica* + (*Nylanderia* + *Lasius*) + (*Anoplolepis* + *Acropyga*))) was recovered.

Formicinae is a large subfamily of Formicidae, comprising about 3000 described species, distributed globally across a wide range of terrestrial environments. This subfamily includes such well-known taxa as weaver ants (*Oecophylla*), wood ants and their relatives (*Formica*), honeypot ants (*Myrmecocystus*), carpenter ants (*Camponotus*) and a diverse array of about fifty other genera (Ward et al. 2016). *Camponotus*, one of the largest and highly evolved genera in Formicinae, and is characterized by an obvious polymorphism and division of labor (Zhou et al. 2018; Xu et al. 2021). *Camponotus japonicus* Mayr, 1866 is one of the most common and widespread ant species in China, and is also a natural enemy for many forest pests and one of the useful medical insects (Zhou et al. 2018). For further study on population genetic structure of *C. japonicus*, we sequenced the complete mitogenome of *C. japonicus* and analyzed the phylogenetic relationships of Formicinae based on mitogenome data.

Male adults of *C. japonicus* were collected from Qingdao City, Shandong Province, China (36°18′N, 120°19′E, July 2020) and were stored deposited in the Entomological Museum of Qinghai University (Accession number QHU-ECJ03, Dr. Wei Li, lwbabylw@163.com). All animal handling and experimental procedures were approved by the Animal Welfare Ethical Committee and the Animal Experimental Ethics Committee of the Qinghai University (Xining, China). Total genomic DNA was extracted from muscle tissues of the thorax using DNeasy DNA Extraction kit (Qiagen, Hilden, Germany). A pair-end sequence library was constructed and sequenced using Illumina HiSeq 2500 platform (Illumina, San Diego, CA), with 150 bp pair-end sequencing method. A total of 28.6 million reads were generated and had been deposited in the NCBI Sequence Read Archive (SRA) with accession number SRR16311555. Raw reads were assembled using MITObim v 1.7 (Hahn et al. 2013). By comparison with the homologous sequences of other Formicidae species from GenBank, the mitogenome of *C. japonicus* was annotated using software GENEIOUS R11 (Biomatters Ltd., Auckland, New Zealand).

The complete mitogenome of *C. japonicus* is 16,422 bp in length (GenBank accession no. OK509076), and contains the typical set of 13 protein-coding, two rRNA and 22 tRNA genes, and one non-coding AT-rich region. Gene order was conserved and identical to most other previously sequenced Formicidae (Babbucci et al. 2014; Kim et al. 2016; Lee et al. 2018; Park et al. 2019). The nucleotide composition of the mitogenome is 77.8% A + T content (A 39.1%, T 38.7%, C 15.6%, G 6.6%). The 22 tRNA genes vary from 54 bp (*trnE*) to 72 bp (*trnH*). Two rRNA genes (*rrnL* and *rrnS*) locate at *trnL1*/*trnV* and *trnV*/control region, respectively. The lengths of *rrnL* and *rrnS* in *C. japonicus* are 1,466 and 765 bp respectively, with AT contents of 84.3% and 85.4%, respectively. Four PCGs (*nad1*, *nad4*, *nad4l* and *nad5*) were encoded by the minority strand (N-strand) while the other nine were located on the majority strand (J-strand). All PCGs of *C. japonicus* have the conventional start codon for invertebrate mitochondrial PCGs (five ATT, four ATA and four ATG). All PCGs terminated with the stop codon TAA.

Phylogenetic analysis was performed based on the nucleotide sequences of 13 PCGs from 19 Formicidae species. Alignments of individual genes were concatenated using SequenceMatrix 1.7.8 (Vaidya et al. 2011). Phylogenetic tree was constructed through raxmlGUI 1.5 (Silvestro and Michalak 2012). Phylogenetic analysis positioned *C. japonicus* in a well-supported clade with the same genus species *C. atrox* and *C. concavus* (Figure 1), and *C. japonicus* was more closely related to *C. atrox* than to *C. concavus*. Then this clade got together with *Polyrhachis dives* and *Colobopsis nipponica*, indicating genus *Camponotus* had a close relationship with *Polyrhachis* and *Colobopsis*. Within Formicinae, the topology (*Colobopsis* + (*Polyrhachis* + *Camponotus*) + (*Formica* + (*Nylanderia* + *Lasius*) + (*Anoplolepis* + *Acropyga*))) was recovered. In conclusion, the mitogenome of *C. japonicus* is decoded in this study and can provide essential and important DNA molecular data for further phylogenetic and evolutionary analysis of Formicidae.

**Figure 1.**
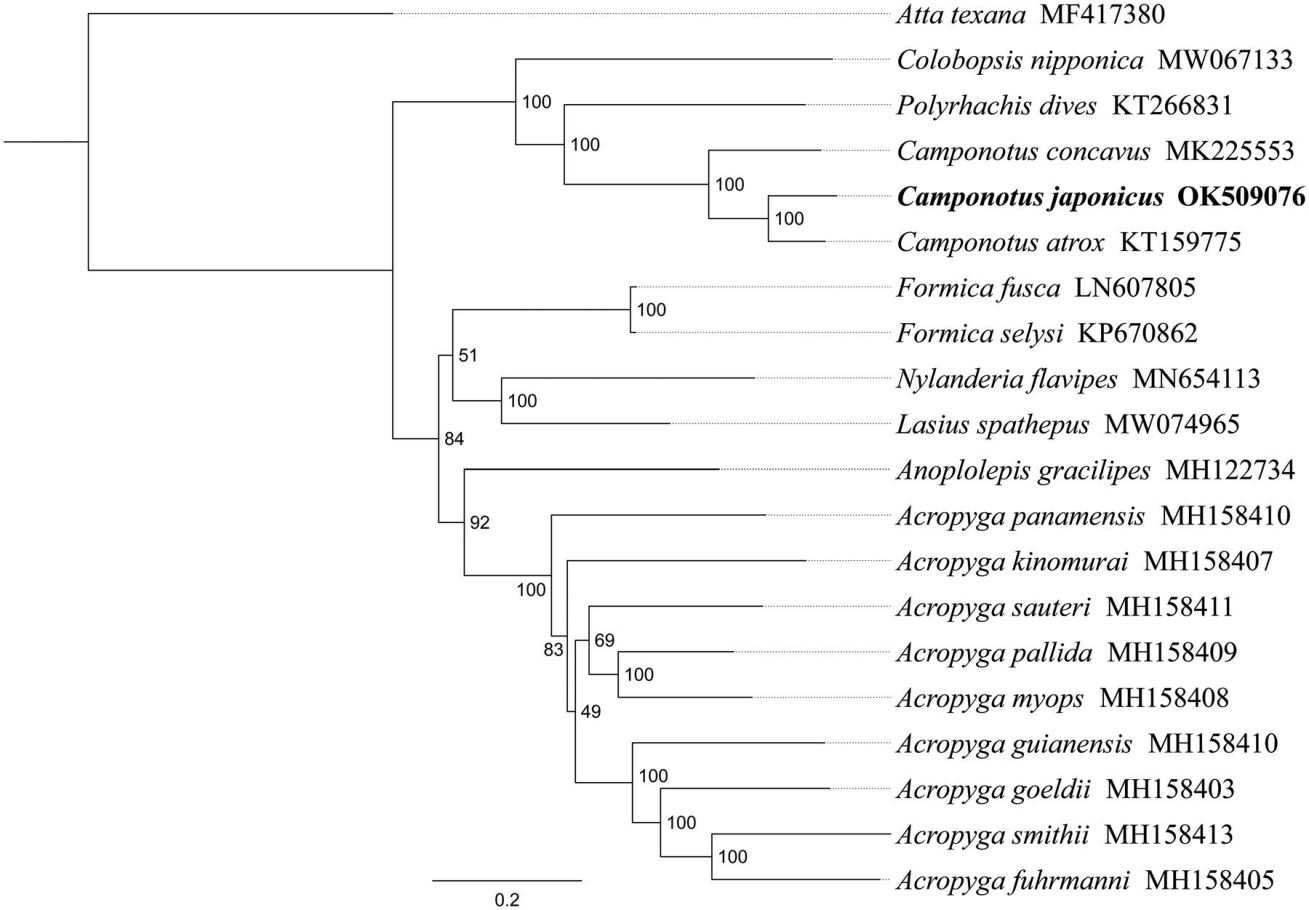
Phylogenetic relationships based on the 13 mitochondrial protein-coding genes sequences inferred from RaxML. Numbers on branches are Bootstrap support values (BS).

## Data Availability

The data that support the findings of this study are openly available in NCBI (National Center for Biotechnology Information) at https://www.ncbi.nlm.nih.gov/, reference number OK509076. The associated BioProject, SRA, and Bio-Sample numbers are PRJNA770858, SRR16311555, and SAMN22242522 respectively.
